# Efficacy of fluoroquinolone prophylaxis in high-risk neutropenic patients with haematological malignancies: a systematic review with meta-analysis

**DOI:** 10.1007/s15010-026-02824-9

**Published:** 2026-05-27

**Authors:** Lorenzo Onorato, Margherita Macera, Mariagrazia Palamone, Francesca Ambrisi, Nicola Coppola

**Affiliations:** Department of Mental Health and Public Medicine- Infectious Diseases Unit, Section of Infectious Diseases, University of Campania Luigi Vanvitelli, Via L. Armanni 5, 80131 Naples, Italy

**Keywords:** Febrile neutropenia, Haematological malignancies, Fluoroquinolone, Prophylaxis, Bloodstream infections

## Abstract

**Objectives:**

To compare the clinical and microbiological outcomes of high-risk neutropenic patients receiving or not fluoroquinolone prophylaxis.

**Study eligibility criteria:**

Randomized controlled trials (RCTs) and observational studies investigating overall mortality and rate of febrile episodes, of bloodstream infections and of isolation of a fluoroquinolone-resistant strain among patients receiving or not prophylaxis.

**Participants:**

Patients with high-risk neutropenia due to haematological malignancies.

**Interventions:**

Prophylaxis with either ciprofloxacin or levofloxacin.

**Comparator:**

No prophylaxis.

**Methods of data synthesis:**

We conducted a meta-analysis pooling risk ratios (RRs) through random effect models.

**Results:**

Among 1731 articles screened, 18 original studies fulfilled the inclusion criteria and were included in the analysis, accounting for a total of 12,586 patients, 7157 (56.9%) of which receiving fluoroquinolones prophylaxis. The analysis assessing the primary outcome including 12,134 patients showed similar mortality rates between groups (RR 1.04, 95% CI 0.79–1.37, *p* = 0.77), with a low statistical heterogeneity observed (I^2^ 49.4%, *p* = 0.022). These findings were confirmed (RR 1.16, 95% CI 0.89–1.50, *p* = 0.27) in the sub-analysis excluding studies conducted in settings with low prevalence of fluoroquinolone resistance, on a total of 3121 patients, and a low heterogeneity was observed (I^2^ 27.6%, *p* = 0.18). Regarding the incidence of febrile episodes, a lower rate was observed among the 1701 patients receiving prophylaxis as compared to the 1514 not receiving prophylaxis (RR 0.87, 95% CI 0.88–0.96, *p* = 0.006); similarly, a lower rate of bloodstream infections was demonstrated among subjects receiving fluoroquinolone prophylaxis (RR 0.59, 95% CI 0.47–0.75, *p* < 0.001) in a meta-analysis including 11,681 patients. Finally, no difference in the isolation rate of fluoroquinolone resistant strains was observed (RR 0.87, 95% CI 0.34–2.20, *p* = 0.77) among 1481 patients included.

**Conclusions:**

Our data suggest that fluoroquinolone prophylaxis in high-risk neutropenic patients is associated with a reduction in the incidence of febrile episodes and bacteraemia but does not provide a survival benefit.

**Supplementary Information:**

The online version contains supplementary material available at 10.1007/s15010-026-02824-9.

## Introduction

Chemotherapy-induced neutropenia and mucositis can predispose patients to invasive infections, most commonly caused by bacteria and fungi [1]. The use of antibacterial prophylaxis with fluoroquinolones to prevent neutropenic fever (NF) and serious infections in patients with hematological malignancies or those undergoing hematopoietic stem cell transplantation (HSCT) remains a current and debated topic, primarily considering the prevalence of multi-drug resistant (MDR) microorganisms in a vision of antimicrobial stewardship program [2].

Several studies have identified risk factors for severe infections in neutropenic patients, enabling clinicians to stratify patients into low- and high-risk categories [3]. It is generally agreed that for low-risk patients — such as those with solid tumors and an expected duration of neutropenia of less than 7 days—fluoroquinolone prophylaxis is not necessary [3, 4]. In contrast, its use in high-risk groups, including patients with acute leukemia or HSCT recipients, remains controversial.

The debate centers around the balance between the potential benefits in preventing serious bacterial infections and the broader consequences of widespread fluoroquinolone use, particularly the emergence of antimicrobial resistance (AMR). The 2018 ASCO/IDSA Clinical Practice Guidelines [3] recommend fluoroquinolone prophylaxis for high-risk patients expected to experience profound, prolonged neutropenia (neutrophil count < 0.1 × 10^9^/L for at least 7 days), such as those with acute myeloid leukemia (AML), myelodysplastic syndromes, or patients undergoing myeloablative conditioning prior to HSCT.

These recommendations differ from the 2011 Australian Consensus Guidelines [5] and the 2016 European Society for Medical Oncology (ESMO) Clinical Practice Guidelines [4], both of which advise against routine fluoroquinolone prophylaxis even in high-risk patients. As early as 2006, Leibovici and colleagues [6] emphasized that, based on the findings of the large and influential study by Bucaneve et al. published in 2005 [7], fluoroquinolone prophylaxis proved effective even in populations where Gram-negative bacteria had resistance rates of around 20%.

In the present meta-analysis we aimed at evaluating impact of fluoroquinolones prophylaxis on clinical outcomes and selection of resistance among high-risk neutropenic patients with hematological malignancies.

## Methods

### Data collection

A systematic literature review of articles on fluoroquinolone prophylaxis in neutropenic patients because of hematological malignancies at high risk of febrile episodes or following HSCT was performed. The study was conducted in accordance with the indication of the PRISMA guidelines [8]. The protocol was registered on PROSPERO database, n. CRD42024520913.

### Search strategy

Two authors (FA, MM) searched MEDLINE and EMBASE for original reports published from January 2015 up to December, 31, 2025; the following items were used for the electronic research: “fluoroquinolones” “ciprofloxacin”, “levofloxacin”, “antibiotic prophylaxis”, “antimicrobial prophylaxis”, “febrile neutropenia”, “haematological malignancies”, “haematopoietic stem cell transplant”. Furthermore, a manual search among the references listed in all retrieved papers to locate any other eligible study. More details on the search strategy are reported in supplementary material.

The following inclusion criteria were used: (a) present original data from experimental and observational studies; (b) compare clinical outcomes of patients receiving fluoroquinolones prophylaxis or not in neutropenic patients with hematological malignancies at high risk of febrile episodes (acute leukemia or multiple myeloma treated with high-dose chemotherapy) or following HSCT; (c) report at least one of the following outcomes: overall mortality, rate of bloodstream infections; rate of episodes of febrile neutropenia, rate of colonization or infection due to fluoroquinolone-resistant strains; (d) include at least 10 patients; (e) be published as a full paper from 2015 to December 2025; (f) be written in English language. We decided to exclude studies published before 2015 because we aimed to collect the most recent evidence on an ever-evolving field. Moreover, a previous large meta-analysis on the same topic [9] enrolled studies published up to 2014.

Papers were excluded if they were: (a) meta-analyses, letters, reviews, meeting abstracts, or editorial comments; (b) duplicate publications; (c) enrolling patients receiving antibacterial prophylaxis other than fluoroquinolones.

Two researchers (FA, MP) independently evaluated the title and abstracts of all citations to select the articles to be included in the analysis, reporting the reasons of exclusion for each study. Thereafter, they retrieved as full texts the articles previously selected to evaluate them for inclusion. In case of disagreement, a third author (LO) took the definite decision.

### Definition and outcomes

The primary outcome evaluated was overall mortality at 1, 6 or 12 months; secondary outcomes included rate of febrile neutropenia episodes and rate of bloodstream infections, both evaluated at 30 days or until neutrophil recovery, and rate of colonization or infection due to fluoroquinolone-resistant strains during follow-up.

Subgroups analyses were conducted analyzing these outcomes in patients enrolled in areas with prevalence of fluoroquinolone resistance among *E*. *coli* > 20% in community or > 30% in hospital setting, as well as among patients receiving hemopoietic stem cell transplantation and among those treated with high dose chemotherapy for acute leukemia. Additional subgroup analyses included only prospective and retrospective studies, only adult and pediatric populations, as well as studies using levofloxacin or ciprofloxacin as prophylaxis. Data on background prevalence of resistance were retrieved either from those provided by the original publications or from independent sources, such as surveillance reports [10].

### Data analysis

Two authors (MM, FA) independently extracted the data using a pre-defined data collection form. The following data were collected for each included paper: last name of the first author, year of publication, country where the study was conducted, study design, sample size, baseline patient characteristics [i.e., median age (IQR), hematological condition, prophylaxis received], median duration of follow-up, and occurrence of the endpoint evaluated in each group.

The quality appraisal of each study was conducted by two reviewers (MM, FA) independently using the Cochrane Risk of Bias Tool version 2 for RCTs [11], and the Newcastle Ottawa scale for observational studies [12]. In the case of disagreement, a third author (LO) decided.

Risk ratios (RRs) were the meta-analytic measure used. We analyzed heterogeneity between the studies using the I². In case of I² <25% no heterogeneity was detected; I^2^ values between 25% and 49% were considered as low heterogeneity, while values between 50% and 75% and over 75% denoted moderate and high heterogeneity, respectively; a P value of Q statistic less than 0.10 was considered significant [13]. Only random-effect size was used. The presence of publication bias was assessed through visual inspection of the funnel plots; an Egger’s test [14] was performed only in meta-analysis including at least 10 studies; a P value of less than 0.10 was considered significant. Where not specified, tests were two-sided, and P values < 0.05 were considered significant. All statistical analyses were performed using Stata/IC, version 16 software (Stata Corporation, College Station, TX, USA).

## Results

### Baseline characteristics of included studies

**Fig. 1:**
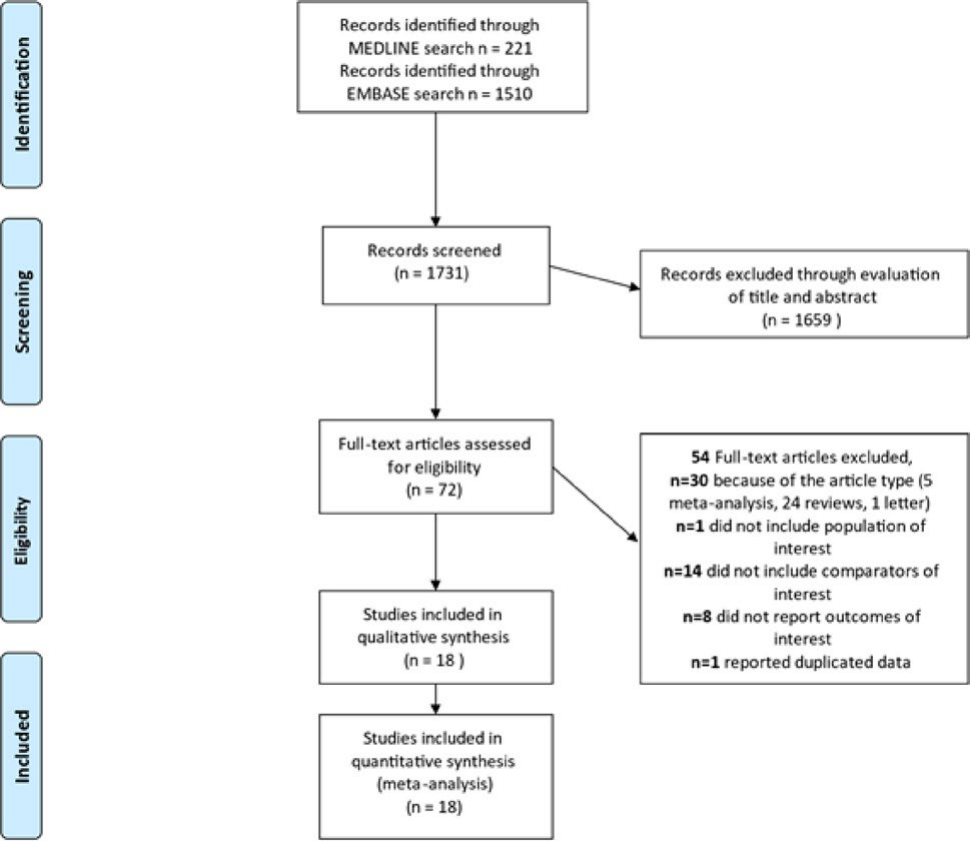
PRISMA flow diagram of the process of identification and selection of articles included in the meta-analysis

After an initial literature search that yielded 1731 articles, 72 original studies were selected for full-text review based on title and abstract. Of these, 18 [15–32] fulfilled the pre-defined inclusion criteria and were included in the final analysis (Fig. 1).

The main characteristics of the included studies are summarized in Table 1. The studies were published between 2015 and 2025 and were conducted across diverse geographical regions, including Europe (*n* = 6), North America (*n* = 5), Asia (*n* = 3), Middle East (*n* = 2), South America (*n* = 1), and North Africa (*n* = 1). The underlying hematologic condition was acute leukemia treated with high dose chemotherapy in 9 studies [15, 16, 23, 25–30], hematopoietic stem cell transplantation (autologous or allogeneic) in 7 [17–19] [22, 24, 31, 32], 1 multiple myeloma [20] and one enrolled both patients with acute leukemia treated with chemotherapy and patients receiving HSCT [21]. Four studies enrolled exclusively pediatric populations [15, 16, 24, 25] while the remaining included adults. Of the 18 included studies, 12 had a retrospective design [16–18, 22–24, 27–32], 3 were prospective cohort studies [21, 25, 26], and 3 were randomized controlled trial [15, 19, 20]. Most studies were single-center, while 6 had a multicenter design [15, 16, 20, 21, 23, 27]. The sample size per study arm ranged from 61 to over 1200 patients.

**Table 1:** Baseline characteristics of studies included in the meta-analysis

First author, year [Reference]	Study design	Country	Sample size	Age (median, IQR)	Indication for prophylaxis (*n*,%)	*N*. patients in prophylaxis group (*n*,%)	Fluoroquinolone used	Fluoroquinolone dose	Febrile neutropenia episodes (*n*, %)	Bloodstream infections episodes (*n*, %)	Mortality (*n*, %)	Isolation of a fluoroquinolone-resistant pathogen (*n*,%)	Median prophylaxis duration (days, range)	Median follow-up duration (range)
Dufrayer, 2023 [15]	RCT	Brazil	20	Prophylaxis n: 9.5 (IQR 2–14.0) Control: 8.0 (IQR 2–13.5);	ALL	10 (50)	Levofloxacin	1 to < 5 y: 10 mg/kg BID; ≥ 5 y: 10 mg/kg once daily (max 750 mg)	9 (45%)	NA	1 (5%)	1 (10%)	29 (23–37)	289 days (27–394)
Clerici, 2023 [16]	Multicenter, retrospective cohort study	Italy	416	6.3 (IQR 3.7–9.9)	ALL	174 (41.8)	Levofloxacin	500 mg QD	177 (42.5)	56 (13.5)	3 (0.7%)	10 (5.7%)	23 (18–31)	12 months (9–15)
Drayson, 2019 [20]	RCT	UK	977	67 (range 59–73)	Multiple myeloma	489 (50.1)	Levofloxacin	500 mg QD	103 (10.5)	66 (6.7)	57 (5.8)	13 (1.3)	84	12 months (IQR 6–24)
Ejaz, 2023 [25]	Single-center prospective cohort study	Pakistan	200	7 (IQR 4–11)	ALL	100 (50)	Levofloxacin	Age < 5 y: 10 mg/kg BID; age ≥ 5 y: 10 mg/kg QD (max 500 mg)	89 (44.5)	30 (15)	8 (4)	9 (4.5)	21 (14–28)	90 days (60–120)
Ghadiany, 2016 [26]	RCT	Iran	80	28 (range 18–45)	AML	40 (50)	Ciprofloxacin	500 mg BID	35 (43.7)	13 (16.2)	4 (5)	3/40 (7.5)	21 (18–28)	60 days (45–90)
Urbino, 2023 [27]	Multicenter, retrospective cohort study	Italy	215	38 (IQR 25–51)	AML	97 (45.1)	Levofloxacin	500 mg QD	111 (51.6)	37 (17.2)	9 (4.2)	7/97 (7.2)	24 (18–30)	90 (60–120)
Kern, 2018 [21]	Multicenter prospective cohort study	Germany, Austria, Switzerland	8,755	Prophylaxis: 57 (IQR 47–64); Control: 57 (IQR 48–65)	AML: 930 (10.6) Allo-HSCT: 4223 (48.2) Auto-HSCT: 3602 (41.1)	5,302 (60.6)	Ciprofloxacin or levofloxacin	Not specified	NA	1358 (15.5)	232 (2.6)	NA	Not specified	10–12 days
Clemmons, 2018 [31]	Single-center, retrospective cohort study	USA	171	59 (range 17–75)	Auto-HSCT: 115 (67.3) Allo-HSCT: 56 (32.7)	105 (61.4)	Levofloxacin	500 mg QD	112 (65.5)	37 (21.6)	6 (3.5)	16/30 (53.3)	8 (1–24)	30 days post-transplant
Ganti, 2017 [28]	Single-center retrospective cohort study	USA	145	Prophylaxis: 58 (range 18–76) Control: 59 (range 22–84)	AML	48 (33.1)	Levofloxacin	500 mg QD	87.5% in prophylaxis vs. 93.8% in control	70 (48.3)	19 (13.1)	NA	19 (5–72)	26 days; (7–78)
Hafez, 2015 [32]	Single centre retrospective cohort study	Egypt	96	4.0 (range 1.1–16.0); Control: 5.0 (1.5–17.0)	Auto-HSCT	50 (52.1)	Levofloxacin	≥ 5 y: 10 mg/kg QD (max 750 mg); <5 y: 10 mg/kg BID (max 500 mg/day)	78 (81.2)	29 (30.2)	2 (2.1)	NA	15	24 days (20–36)
Tabarraee, 2016 [17]	Single-center retrospective cohort study	Iran	72	Prophylaxis: 37.4 (range 22–52) Control 39.7 (range 25–53)	Auto-HSCT	30 (41.7)	Ciprofloxacin	500 mg BID	59 (81.9)	15 (20.8)	NA	NA	8.1 (3–12)	18.2 days (13–23) in prophylaxis group; 22.2 (13–31) in control
Yeshurun, 2018[18]	Single center retrospective cohort study	Isarel	356	58 (range 19–72)	Auto-HSCT	177 (49.7)	Ciprofloxacin	500 mg BID	308 (86.5)	35 (9.8)	6 (1.7)	10 (28.6)	7 (4–14)	30 days post-transplant
Alexander, 2018 [19]	RCT	USA	171	45 (range 18–67)	Allo-HSCT	102 (59.6)	Levofloxacin	> 6 months to < 5 y: 10 mg/kg BID; > 5 y: 10 mg/kg QD (max 750 mg)	104 (60.8)	36 (21)	3 (1.7)	NA	17 (10–28)	100 days (90–120)
Stern, 2024 [22]	Single-center retrospective, cohort study	Israel	289	59 (range 22–73)	Auto-HSCT	150 (50.3)	Ciprofloxacin or levofloxacin	Ciprofloxacin: 500 mg BID; Levofloxacin: not specified	227 (78.5)	57 (19.7)	5 (1.7)	11/57 (19.3)	10 (7–17)	30 days post-transplant
Caro, 2022 [29]	Single-center retrospective cohort study	USA	121	64 (IQR 52–71)	AML	87 (71.9)	Levofloxacin	Not specified	84 (69.4)	27 (31)	65(53.7)	6 (4.9)	10 (IQR 6–22)	37.8 months (IQR 20.8–65.4)
Lee, 2018 [30]	Single-center retrospective, cohort study	Canada	100	Prophylaxis: 52.6 Control: 49.8	AML	50 (50)	Levofloxacin	500–750 mg QD	57 (57)	NA	NA	6/13 (46.1)	10 (7–14)	30 days post-discharge
Brousse, 2025 [23]	Multicenter retrospective cohortstudy	France	258	69.8 (IQR 59.7–75.4)	AML treated with venetoclax + azacitidine	72 (27.9)	Levofloxacin	500 mg/day from day 10 after first VEN-AZA course until neutrophil recovery > 0.5 × 10^9/L	24 (33.3)	6 (8.3)	0 (0.0)	5 (20.8)	20 days (IQR 15–40)	56 days after first cycle
Leardini, 2025 [24]	Single-center retrospective cohort study	Italy	143	9 years (range 0.3–23.0)	Allo- HSCT	74 (51.4)	Levofloxacin	10 mg/kg/day IV	NA	15 (20.3)	7 (9.5)	12 (80.0)	From conditioning to engraftment	100 days after HSCT

*RCT *randomized controlled trial, *ALL* acute lymphoblastic leukemia, *AML* acute myeloid leukemia, *HSCT* hematopoietic stem cell transplantation

Levofloxacin was used as prophylaxis in 13 studies [15, 16, 19, 20, 23–25, 27–32], ciprofloxacin in 3 studies [17, 18, 26], while 2 studies [21, 22] included patients receiving levofloxacin or ciprofloxacin. Prophylaxis generally started at the beginning of chemotherapy or conditioning and continued until neutrophil recovery, fever onset, or hospital discharge. The median duration of prophylaxis ranged from 7 to 37 days. The duration of follow-up ranged from 21 to 365 days. In 9 studies [18, 19, 22, 24–27, 30, 31], outcomes were assessed at a fixed timepoint (most commonly day + 30 or + 100), while in 5 [17, 21, 23, 28, 32] studies follow-up extended until neutrophil recovery or end of hospitalization. Only 4 studies [15, 16, 20, 29] included longer follow-up periods (6–12 months). Eleven studies [18, 20–29] reported data on all-cause mortality, while two additional studies [16, 32] reported the rate of infection-related mortality. The rate of bloodstream infections due to fluoroquinolone-resistant strains was reported in 4 studies [23, 24, 27, 29], while 2 additional studies [18, 30] reported the rate of any type of infections due to fluoroquinolone-resistant strains.

### Quality appraisal of included studies

The quality appraisal of the three RCTs included in the analysis is displayed in supplementary Table 1; one study [20] was judged as having a low risk of bias, one [19] as having some concerns, while the last RCT [15] was deemed at high risk of bias.

Regarding observational studies (supplementary Table 2), 8 out of 15 [17, 21, 23–26, 30, 32] were considered at intermediate risk of bias, while 7 [16, 18, 22, 27–29, 31] were judged at low risk of bias.

### Meta-analysis of studies evaluating mortality rate in patients receiving or not receiving fluoroquinolones prophylaxis

**Fig. 2:**
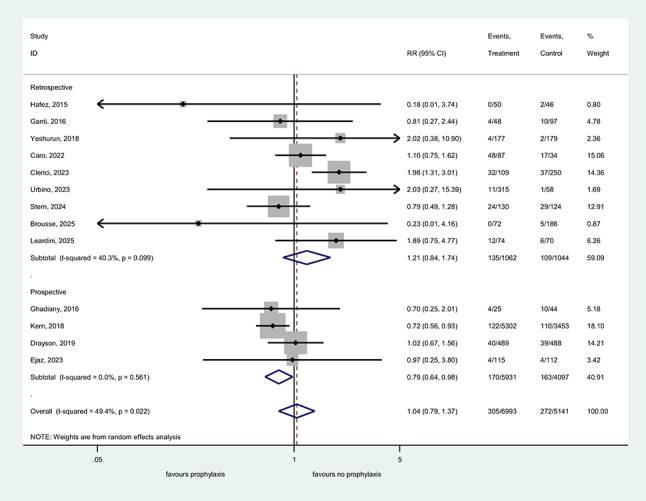
Meta-analysis of studies comparing mortality rate in patients receiving or not receiving fluoroquinolone prophylaxis

The meta-analysis evaluating the primary outcome, overall mortality, included 13 studies [16, 18, 20–29, 32], for a total of 12,134 patients, 6993 (57.6%) in the prophylaxis group and 5141 (42.4%) not receiving prophylaxis (Table 2; Fig. 2). The analysis showed similar mortality rate between groups (RR 1.04, 95% CI 0.79–1.37, *p* = 0.77), with a low statistical heterogeneity observed (I^2^ 49.4%, *p* = 0.022), while no evidence of publication bias emerged from the visual inspection of funnel plots (Supplementary Fig. 2) and Egger’s test (*p* = 0.77). Same results were observed among the 9 retrospective studies [16, 18, 22–24, 27–29, 32] accounting for 2,106 patients (RR 1.21, 95% CI 0.84–1.74, *p* = 0.29), while the four prospective studies [20, 21, 25, 26] showed a lower mortality rate among the 5,931 patients receiving prophylaxis (RR 0.79, 95% CI 0.64–0.98, *p* = 0.03). When we excluded the 2 studies [21, 23] conducted in a setting with low prevalence for fluoroquinolone resistance, these findings were confirmed (RR 1.16, 95% CI 0.89–1.50, *p* = 0.27), and a low heterogeneity was observed between studies (I^2^ 27.6%, *p* = 0.18) (Supplementary Figs. 1 and 2). Similar results were obtained in the sub-analysis including only patients receiving HSCT (RR 1.25, 95% CI 0.81–1.92, *p* = 0.31) enrolled in 6 studies [16, 18, 21, 22, 24, 32], as well as in the analysis including the 7 studies [21, 23, 25–29] enrolling the 1,846 patients treated with high dose chemotherapy for acute leukemia (RR 0.77, 95% CI 0.48–1.22, *p* = 0.27) (Supplementary Figs. 1 and 2). When we limited the analysis to the 9 studies [16, 20, 23–25, 27–29, 32] enrolling patients who received levofloxacin as prophylaxis, no difference in mortality emerged among groups (RR 1.26, 95% CI 0.94–1.68, *p* = 0.12). The same findings were observed among the 11,667 adult patients included in 10 studies [16, 18, 20–23, 26–29] (RR 1.02, 95% CI 0.76–1.37, *p* = 0.91), as well as in the 466 pediatric patients enrolled in 3 studies [24, 25, 32] (RR 1.25, 95% CI 0.51–3.04, *p* = 0.62).

**Table 2:** Meta-analysis of studies comparing clinical and microbiological outcomes of patients receiving or not receiving fluoroquinolone prophylaxis

Outcome	*N*° of studies [References]	*N*° of patients in prophylaxis/no prophylaxis group	*N*° of events in prophylaxis (%)/no prophylaxis (%) group	RR	95% CI	*p*	Heterogeneity test (I^2^,%; *p*)
Mortality Prospective studies Retrospective studies	13 [16, 18, 20–29, 32] 4 [20, 21, 25, 26] 9 [16, 18, 22–24, 27–29, 32]	6993/5141 5931/4097 1062/1044	305 (4.4)/272 (5.3) 170 (2.9)/163 (4.0) 135 (12.7)/109 (10.4)	1.04 0.79 1.21	0.79–1.37 0.64–0.98 0.84–1.74	0.775 0.03 0.296	49.4;0.022 0.0;0.561 40.3;0.096
Mortality (high prevalence of fq resistance)	11 [16, 18, 20, 22, 24–29, 32]	1619/1502	183 (11.3)/157 (10.5)	1.16	0.89–1.50	0.275	27.6;0.18
Mortality (HSCT recipients)	6 [16, 18, 21–29, 31]	3715/2935	143 (3.8)/121 (4.2)	1.25	0.81–1.92	0.309	58.3;0.035
Mortality (Acute Leukemia patients)	7 [21, 23, 25–29]	1051/795	81 (7.7)/66 (8.3)	0.77	0.48–1.22	0.267	33.9;0.17
Mortality (patients receiving levofloxacin)	9 [16, 20, 23–25, 27–29, 32]	1359/1341	151 (11.1)/121 (9.0)	1.26	0.94–1.68	0.124	24.4;0.23
Mortality (adult patients)	10 [16, 18, 20–23, 26–29]	6754/4913	289 (4.3)/260 (5.3)	1.02	0.76–1.37	0.913	56.2;0.015
Mortality (pediatric patients)	3 [24, 25, 32]	238/228	16 (6.7)/12 (5.3)	1.25	0.51–3.04	0.623	18.4;0,294
Febrile neutropenia events Prospective studies Retrospective studies	12 [15, 17–19, 22, 23, 25–27, 29–31] 4 [15, 19, 25, 26] 8 [17, 18, 22, 23, 27, 29–31]	1701/1514 678/725 1023/789	1029 (60.1)/885 (57.7) 273 (40.3)/348 (48) 756 (73.9)/537 (68)	0.88 0.82 0.90	0.81–0.96 0.65–1.05 0.81–0.99	0.006 0.12 0.028	70.5;<0.001 63.1;0.04 73.2;<0.001
Febrile neutropenia events (only HSCT recipients)	6 [15, 25–27, 29, 30]	798/764	577 (72.3)/646 (84,5)	0.83	0.71–0.96	0.028	86.6;<0.001
Febrile neutropenia events (only Acute Leukemia patients)	7 [15, 23, 25–27, 29, 30]	731/544	470 (64.3)/279 (51.3)	0.85	0.70–1.03	0.106	76.8;<0.001
Febrile neutropenia events (patients receiving levofloxacin)	8 [15, 19, 23, 25, 27, 29–31]	1339/1225	725 (54.1)/550 (48.9)	0.81	0.70–0.95	0.009	77.4;<0.001
Febrile neutropenia events (patients receiving ciprofloxacin)	3 [17, 18, 26]	232/265	191 (82.7)/232 (88.3)	0.93	0.87–1.00.87.00	0.063	0.0;0.872
Febrile neutropenia events (adult patients)	9 [17, 18, 22, 23, 26, 27, 29–31]	1048/833	776 (73.9)/573 (68)	0.90	0.82–0.99	0.028	69.4:0.001
Febrile neutropenia events (pediatric patients)	3 [15, 19, 25]	653/681	253 (38.7)/312 (45.8)	0.74	0.49–1.49–12.49	0.154	71.4;0.03
Bloodstream infections rate Prospective studies - Retrospective studies	15 [17–19, 21–32] 4 [19, 21, 25, 26] 11 [17, 18, 22–24, 27–32]	6943/4918 5748/3916 1195/1002	1057 (15.3)/936 (19.0) 839 (14.6)/674 (17.2) 218 (18.2)/262 (26.1)	0.59 0.66 0.57	0.47–0.75 0.42–1.04 0.46–0.70	**< **0.001 0.073 **< **0.001	68.0;<0.001 76.3;0.005 26.1;0.19
Bloodstream infections rate (high prevalence of fq resistance)	13 [17–19, 22, 24–32]	1569/1279	265 (16.9)/344 (26.9)	0.55	0.46–0.67	**<** 0.001	26.1;0.18
Bloodstream infections rate (only HSCT recipients)	8 [17–19, 21, 22, 24, 31, 32]	3951/2801	598 (15.1)/554 (19.8)	0.59	0.43–0.82	0.002	76.9;<0.001
Bloodstream infections rate (only Acute Leukemia patients)	8 [21, 23, 25–30]	1158/895	190 (16.4)/157 (17.5)	0.68	0.54–0.85	0.002	8.4;0.36
Bloodstream infections rate (patients receiving levofloxacin)	10 [19, 23–25, 27–32]	1279/1076	218 (17)/240 (22.3)	0.61	0.51–0.72	**< **0.001	0.0;0.52
Bloodstream infections rate (patients receiving ciprofloxacin)	3 [17, 18, 26]	232/265	12 (5.2)/45 (16.9)	0.37	0.11–1.29	0.12	62.0;0.07
Bloodstream infections rate (adult patients)	11 [17, 18, 21–23, 26–31]	6398/4383	979 (15.3)/805 (18.4)	0.59	0.44–0.78	**<** 0.001	71.1;<0.001
Bloodstream infections rate (pediatric patients)	4 [19, 24, 25, 32]	545/535	78/131	0.61	0.42–0.88	0.008	40.8;0.17
Isolation of fq-resistant strain	6 [18, 23, 24, 27, 29, 30]	832/649	86 (10.3)/60 (9.2)	0.87	0.34–2.20	0.77	78.0;<0.001
Isolation of fq-resistant strain (patients receiving levofloxacin)	5 [23, 24, 27, 29, 30]	655/470	78 (11.9)/33 (7.0)	1.15	0.47–2.85	0.76	66.9;0.017
Isolation of fq-resistant strain (adult patients)	5 [18, 23, 27, 29, 30]	758/579	74 (9.8)/55 (9.5)	0.71	0.25–2.01	0.517	78.3;0.001

*Fq * fluoroquinolone, *HSCT *haematopoietic stem cell transplantation

### Meta-analysis of studies evaluating the rate of febrile episodes in patients receiving or not receiving fluoroquinolones prophylaxis

**Fig. 3:**
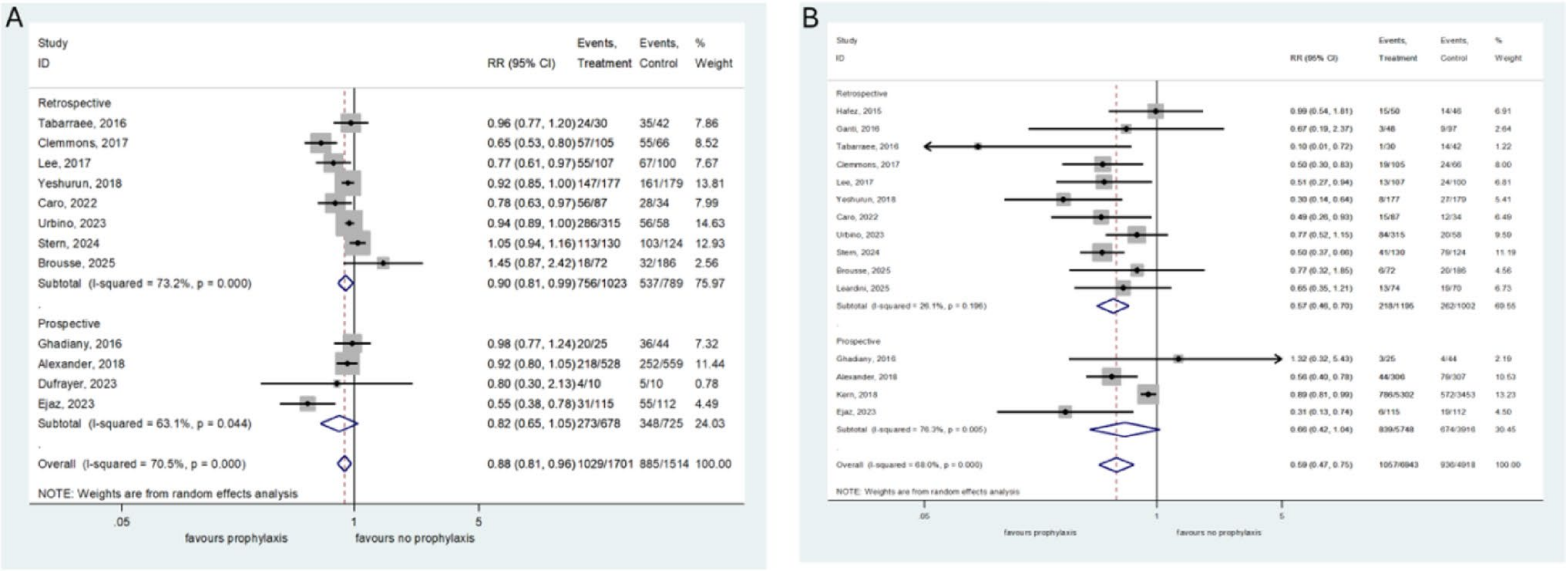
Meta-analysis of studies comparing rate of febrile episodes (**A**) and rate of bloodstream infections (**B**) in patients receiving or not receiving fluoroquinolone prophylaxis

Regarding the incidence of febrile episodes (Table 2), a lower rate was observed among patients receiving prophylaxis (RR 0.87, 95% CI 0.88–0.96, *p* = 0.006), in a meta-analysis including 12 studies [15, 17–19, 22, 23, 25–27, 29–31], for a total of 1701 patients in the prophylaxis group and 1,514 in non-prophylaxis group (Table 2; Fig. 3a). A significant heterogeneity emerged between studies (I^2^ 73.2, *p* < 0.001). Similar results were observed among the 1812 patients enrolled in 8 retrospective studies [17, 18, 22, 23, 27, 29–31] (RR 0.90, 95% CI 0.81–0.99, *p* = 0.62), but not among the 1403 patients enrolled in 4 prospective studies [15, 19, 25, 26]. No sub-analysis was conducted according to the baseline prevalence of resistance since none of the included studies were carried out in a low prevalence area. In a sub-analysis including 1562 patients receiving HSCT enrolled in 6 studies [15, 25–27, 29, 30] a lower incidence of events was detected in the prophylaxis group (RR 0.83, 95% CI 0.71–0.96, *p* = 0.028); conversely, no significant difference was observed among groups in the analysis including only the 1,275 patients with acute leukemia enrolled in 7 studies [15, 23, 25–27, 29, 30] (RR 0.85, 95% CI 0.70–1.03, *p* = 0.106) (Supplementary Figs. 3). Considering the fluoroquinolone used, a lower rate of febrile episodes was detected in the sub-analysis of 8 studies evaluating the efficacy of levofloxacin [15, 19, 23, 25, 27, 29–31] enrolling 2564 patients (RR 0.81, 95% CI 0.70–0.95, *p* = 0.009), although a significant statistical heterogeneity emerged among studies (I^2^ 77.4, *p* < 0.001), while no difference among groups was observed in the sub-analysis of 3 studies [17, 18, 26] using ciprofloxacin as prophylaxis (RR 0.93, 95% CI 0.87–1.00.87.00, *p* = 0.063). The lower rate of events was confirmed in the sub-analysis including 1,891 adult patients enrolled in 9 studies [17, 18, 22, 23, 26, 27, 29–31] (RR 0.90, 95% CI 0.82–0.99, *p* = 0.028), but not among the 3 studies [15, 19, 25] including 1334 pediatric patients (RR 0.81, 95% CI 0.49–1.12, *p* = 0.009).

### Meta-analysis of studies evaluating the rate of bloodstream infections and isolation of a fluoroquinolone resistant strain in patients receiving or not receiving fluoroquinolones prophylaxis

When we analyzed the rate of bloodstream infections, among 15 studies [17–19, 21–32] including 6943 patients in prophylaxis group and 4918 in the control group, a lower rate was demonstrated among subjects receiving fluoroquinolone prophylaxis (RR 0.59, 95% CI 0.47–0.75, *p* < 0.001), with a significant heterogeneity between studies (I^2^ 68.0, *p* < 0.001) (Table 2; Fig. 3b). These findings were confirmed among the 11 retrospective studies[17, 18, 22–24, 27–32] (RR 0.57, 95% CI 0.46–0.70, *p* < 0.001), but not in the 4 prospective studies [19, 21, 25, 26] enrolling 9664 patients (RR 0.66, 95% CI 0.42–1.04, *p* = 0.073).

Again, when excluding the 2 studies carried out in a low prevalence setting for resistance [21, 23], the results were confirmed (RR 0.55, 95% CI 0.46–0.67, *p* < 0.001), and a low heterogeneity was observed (I^2^ 26.1, *p* = 0.18). The same findings were described when including the 6,752 HSCT recipients enrolled in 8 studies [17–19, 21, 22, 24, 31, 32] (RR 0.589 95% CI 0.43–0.82, *p* = 0.002), and when analyzing the outcome of 2053 patients treated for acute leukemia in 8 studies [21, 23, 25–30] (RR 0.68, 95% CI 0.54–0.85, *p* = 0.002) (Supplementary Fig. 5). Similarly, a lower rate of events was observed in the sub-analysis of 10 studies [19, 23–25, 27–32] evaluating the impact of levofloxacin prophylaxis (RR 0.61, 95% CI 0.51–0.72, *p* < 0.001), with no statistical heterogeneity detected (I^2^ 0.0, *p* = 0.52), while the results were no confirmed in the meta-analysis of 3 studies using ciprofloxacin [17, 18, 26] (RR 0.37, 95% CI 0.11–1.29, *p* = 0.12). A lower rate of bloodstream infections was also observed in the prophylaxis group among the 10,781 adult patients enrolled in 11 studies [17, 18, 21–23, 26–31] (RR 0.59, 95% CI 0.44–0.78, *p* < 0.001), as well as among the 1,080 pediatric subjects enrolled in 4 studies [19, 24, 25, 32] (RR 0.61, 95% CI 0.42–0.88, *p* = 0.008).

Finally, in a meta-analysis including 6 studies [23], 2418, [27, 29, 30], for a total of 1,481 patients, no difference in the isolation rate of fluoroquinolone resistant strains was observed (RR 0.87, 95% CI 0.34–2.20, *p* = 0.77), with significant heterogeneity (I^2^ 78.0, *p* < 0.001) among studies (supplementary Fig. 7). The same results were observed in the 5 studies [23, 24, 27, 29, 30] including patients who received levofloxacin prophylaxis (RR 1.15, 95% CI 0.47–2.85, *p* = 0.76), as well as among the 5 studies [18, 23, 27, 29, 30] enrolling 1,337 adult patients (RR 0.71, 95% CI 0.25–2.01, *p* = 0.52).

## Discussion

Fluoroquinolones prophylaxis in high-risk neutropenic patients remains a debated topic; despite the evidence demonstrating an impact on febrile episode and infectious events, several literature data reported no survival benefits [6, 9]. Moreover, the spread of fluoroquinolones-resistant strains in many geographic areas could undermine the efficacy of prophylaxis, not to mention the potential impact of fluoroquinolone consumption on the selection of multidrug-resistant pathogens.

The results of the present meta-analysis confirm that fluoroquinolone prophylaxis in patients with hematologic malignancies treated with high-dose chemotherapy or undergoing HSCT is associated with a significant reduction in febrile episodes (*p* = 0.006) and bacteremia (*p* < 0.001). However, this benefit does not translate into an improvement in overall survival (RR 1.04; *p* = 0.77). Regarding infections or colonization with resistant strains, the available data are heterogeneous and do not allow for definitive conclusions. When we stratified the patients according to the hematological condition that required prophylaxis administration, the same results were confirmed, with the only exception represented by patients with acute leukemia not receiving HSCT, who did not benefit from prophylaxis in term of significant reduction of febrile episodes. Similarly, most of the results obtained in the general analysis were confirmed in the sub-analyses including adult populations or patients receiving levofloxacin as prophylaxis; however, in our meta-analysis pediatric patients or subjects receiving ciprofloxacin were underrepresented, so no clear evidence can be drawn on these populations.

Our findings are consistent with the results of a previous meta-analysis [9] published in 2018, demonstrating the efficacy of fluoroquinolones prophylaxis in reducing the occurrence febrile episodes and bloodstream infections, without any impact on overall mortality. To interpret this data, we should take into account that the efficacy of prophylaxis is mainly limited to infectious events due to drug-susceptible strains, while one of the most important drivers of mortality in this population is represented by drug-resistance pathogens, leading to higher risk of inadequate empirical antimicrobial treatment [33]. Therefore, fluoroquinolone prophylaxis could have a limited impact on patients’ survival, despite the significant reduction of the incidence of bloodstream infections due to susceptible pathogens. Through a meta-regression analysis, the authors [9] showed no impact of background prevalence of resistance to fluoroquinolones on the efficacy of prophylaxis, although resistance rates were quite limited in most studies, ranging from < 1 to 20% in community and from 1 to 28% in hospital settings.

The meta-analysis conducted by Mikulska [9] et al. included studies published up to 2014; since then, the epidemiology of MDR pathogens and the prevalence of fluoroquinolone resistance have undergone a significant change. In the present meta-analysis, to evaluate the role of fluoroquinolones prophylaxis in settings with high prevalence of resistance we decided to conduct a sub-analysis excluding studies carried in low prevalence area, obtaining similar findings with a significant reduction in statistical heterogeneity. However, we should consider that in some geographic areas the prevalence of fluoroquinolone resistance among Enterobacterales is far higher than 20%; the 2024 data from the EARS-Net [10] has reported a 34.5% resistance to fluoroquinolones among E. coli and 47.7% among K. pneumoniae in Italy, with peaks of resistance rate > 60% in regional reports from Southern Italy [34]. These considerations raise some concern about the possibility of applying our findings in all epidemiological settings.

Since both epidemiology of antimicrobial resistance and therapeutic management of hematological malignancies have significantly changed over time, we decided to focus solely on studies published during last decade; therefore, none of the studies included in our analysis were included in the previous meta-analysis [9]; nevertheless, the same findings were observed, strengthening the reliability of our conclusion. Moreover, since previous meta-analyses have not investigated the impact of prophylaxis in different hematological settings, we analyzed separately patients treated with high dose chemotherapy for acute leukemia and HSCT recipients, reporting no clear benefit of prophylaxis on the occurrence of febrile events in the first group. These results are consistent with the findings of a recent Italian study [27], including only patients with acute leukemia, demonstrating no reduction in the rate of febrile neutropenia and bloodstream infections in subjects receiving prophylaxis.

The topic of the present meta-analysis seems to be important, since a particularly relevant and debated aspect concerns the impact of prophylaxis on the selection of resistant strains. A meta-analysis including 291 papers [35] demonstrated that the exposure to fluoroquinolones is associated with a significant increase in the rate of colonization or infection due to multidrug-resistant bacteria (OR 1.78 95% CI 1.68–1.88). In studies conducted in settings with low resistance prevalence, no significant increase in colonization or infection with multidrug-resistant organisms was observed [20, 21]. Even in more complex epidemiological scenarios, such as those with high resistance rates, prophylaxis was not associated with a statistically significant increase in infections caused by fluoroquinolone-resistant strains, although subgroup heterogeneity and relatively small numbers limit the robustness of the conclusions [22, 29]. Notably, some analyses suggested that even in areas with high resistance prevalence, prophylaxis in hematological patients may reduce the risk of febrile neutropenia and systemic infections without leading to a measurable increase in resistance, although prospective randomized trials are needed for confirmation [29]. In our meta-analysis, no significant difference was observed in terms of infections caused by resistant strains (RR 0.87; *p* = 0.77); however, only 6 studies reported this outcome; the limited sample size, as well as the high statistical heterogeneity among studies, lower the certainty on these findings and do not allow us to rule out a long-term impact of prophylaxis on the epidemiology of resistance. Moreover, the extensive use of fluoroquinolones has not only been associated to a rise in prevalence of multi-drug resistant strains but also to several adverse drug reactions, including Clostridioides difficile infections, musculoskeletal and neuropsychiatric adverse events, arrhythmias, aortic dissection and aneurysm rupture [36]. Thus, safety concerns should be carefully evaluated while balancing pros and cons of fluoroquinolones prophylaxis in hematological patients.

Recommendations issued by scientific societies have never been entirely consistent. The 2018 ASCO/IDSA guidelines [3] remain favorable in patients with profound and prolonged neutropenia, such as those with acute myeloid leukemia or myelodysplastic syndrome, or undergoing myeloablative conditioning before HSCT. In contrast, the 2011 Australian guidelines [5] and the 2016 ESMO guidelines [4] take a more cautious stance, advising against routine use and instead emphasizing prevention strategies and early infection management. Our results fit into this context: they confirm the reduction in fever and bacteremia but show no impact on mortality. Therefore, the issue remains open, and decisions on prophylaxis should be made on a case-by-case basis, considering the type of patient and, most importantly, the local resistance epidemiology.

This meta-analysis has several limitations that must be acknowledged. First, there is significant heterogeneity among the included studies: of the 18 analyzed, most were retrospective, with inherent limitations in terms of selection bias and data completeness. Another issue concerns follow-up, which was not uniformly reported: 17 studies specified it clearly, but in 9 cases it was set at 30 or 100 days, while in 8 others it ended with neutropenia recovery or hospital discharge. This variability makes comparisons more difficult and may have particularly influenced the assessment of mortality, which would require more standardized follow-up. Nevertheless, the large sample size, the availability of meaningful outcomes, such as mortality and incidence of bloodstream infections in most studies, as well as the possibility to stratify the results according to the baseline prevalence of resistance, the underlying hematological condition, the fluoroquinolone used, the type of population and the study design strengthen the findings of our analysis.

## Conclusions

The results of this meta-analysis suggest that fluoroquinolone prophylaxis in patients with hematologic malignancies or undergoing stem cell transplantation is associated with a reduction in the incidence of febrile episodes and bacteremia but does not provide a survival benefit. The impact on the selection of resistant strains remains uncertain: the available data are heterogeneous and do not entirely exclude a long-term effect on resistance epidemiology. In light of these results, prophylaxis should be considered on a case-by-case basis, taking into account the local epidemiological context. Further studies, particularly large multicenter randomized trials, will be needed to better define the balance between clinical efficacy and resistance risk, thereby providing stronger guidance for future recommendations.

## Supplementary Information

Below is the link to the electronic supplementary material.

Supplementary Material 1

Supplementary Material 2

Supplementary Material 3

## Data Availability

No datasets were generated or analysed during the current study.
