# Supplementary material for: Efficacy of fluoroquinolone prophylaxis in high-risk neutropenic patients with haematological malignancies: a systematic review with meta-analysis
Source: Infection. 2026 May 27;54(4):1969–80. doi: 10.1007/s15010-026-02824-9 (PMC13476321; doi:10.1007/s15010-026-02824-9)
Supplement: Supplementary file 2 [file 15010_2026_2824_MOESM3_ESM.docx]

**Search strategy for MEDLINE database:**

("fluoroquinolones" or "ciprofloxacin" or "levofloxacin" or "antibiotic prophylaxis" or “antimicrobial prophylaxis”) and ("febrile neutropenia" or "haematological malignancies" or "Haematopoietic stem cell transplant")

**Search strategy for EMBASE database:**

("fluoroquinolones" or "ciprofloxacin" or "levofloxacin" or "antibiotic prophylaxis" or “antimicrobial prophylaxis”) and ("febrile neutropenia" or "haematological malignancies" or "Haematopoietic stem cell transplant")

|  | **First author, year, [Reference n.]** | Alexander, 2018 [19] | Drayson, 2019 [20] | Dufrayer, 2023 [15] |
| --- | --- | --- | --- | --- |
| **Domain 1: Randomization process** | **1.1 Random sequence generation** | Y | Y | Y |
|  | **1.2 Allocation concealment** | Y | Y | N |
|  | **1.3 Baseline imbalance** | N | N | N |
|  | **Risk of bias judgement** | Low Riak | Low Risk | High risk |
| **Domain 2: Deviations from intended interventions** | **2.1 Patients’ awareness about intervention** | Y | N | Y |
|  | **2.2 Carers’ awareness about intervention** | Y | N | Y |
|  | **2.3 Deviations from intended intervention because of trial context** | PN | NA | N |
|  | **2.4 Deviations likely to affect the outcome** | NA | NA | NA |
|  | **2.5 Deviations balanced between groups** | NA | NA | NA |
|  | **2.6 Appropriateness of analysis** | Y | Y | Y |
|  | **2.7 Impact of failure to analyse participants in the group to which they were randomized** | NA | NA | NA |
|  | **Risk of bias judgement** | Low Risk | Low Risk | Low Risk |
| **Domain 3: Missing outcome data** | **3.1 Data available for all participants** | PN | PY | Y |
|  | **3.2 Evidence that the results were not biased** | PN | NA | NA |
|  | **3.3 Could missingness depend on its true value?** | PY | NA | NA |
|  | **3.4 Is it likely missingness depend on its true value?** | PN | NA | NA |
|  | **Risk of bias judgement** | Some concerns | Low Risk | Low Risk |
| **Domain 4: Measurement of the outcome** | **4.1 Methods of outcomes measurement inappropriate** | N | N | N |
|  | **4.2 Different methods among groups** | N | N | N |
|  | **4.3 Outcomes assessors awareness of intervention** | Y | N | Y |
|  | **4.4 Outcome assessment influenced by knowledge on intervention** | N | NA | N |
|  | **4.5 Outcome assessment likely to be influenced by knowledge** | NA | NA | NA |
|  | **Risk of bias judgement** | Low Risk | Low Risk | Low risk |
| **Domain 5: Selection of the reported results** | **5.1 Pre-specified analysis plan** | N | Y | N |
|  | **5.2 Results selected from multiple outcome measures** | NI | N | NI |
|  | **5.3 Results selected from multiple analysis of the data** | NI | N | NI |
|  | **Risk of bias judgement** | Some concerns | Low Risk | Some concerns |
| **Overall judgement** | **Risk of bias judgement** | Some concerns | Low Risk | High Riak |

**Supplementary Table 1**. Quality assessment of RCTs included in the meta-analysis

| **First author, year, [Reference n.]** | **Selection** | | | | **Comparability** | **Outcomes** | | | **Total score** |
| --- | --- | --- | --- | --- | --- | --- | --- | --- | --- |
|  | **Representativeness**  **of the exposed**  **cohort** | **Selection of**  **the nonexposed**  **cohort** | **Ascertainment**  **of**  **exposure** | **Outcome of interest**  **not present at**  **start of study** | **Comparability**  **based on design**  **or analysis** | **Assessment**  **of**  **outcome** | **Was follow-up**  **long enough for**  **outcomes to occur?** | **Adequacy**  **of follow-up**  **of cohorts** |  |
| **Brousse, 2025 [23]** |  | * | * | * | * | * | * |  | 6* |
| **Caro, 2022 [29]** |  | * | * | * | * | * | * | * | 7* |
| **Clemmons, 2017 [31]** | * | * | * | * |  | * | * | * | 7* |
| **Clerici, 2022 [16]** | * | * | * |  | ** | * | * | * | 8* |
| **Ejaz, 2023 [25]** |  | * | * | * | * | * |  | * | 6* |
| **Ganti, 2016 [28]** |  | * | * | * | ** | * | * | * | 8* |
| **Ghadiany, 2016 [26]** |  | * | * | * |  | * | * |  | 5* |
| **Hafez, 2015 [32]** |  | * | * |  | * | * |  |  | 4* |
| **Kern, 2018 [21]** | * | * | * | * |  | * | * |  | 6* |
| **Leardini, 2025 [24]** |  |  | * | * | ** | * | * |  | 6* |
| **Lee, 2017 [30]** | * | * | * |  |  | * |  |  | 4* |
| **Stern, 2024 [22]** | * | * | * | * | * | ** | * |  | 8* |
| **Tabareee, 2016 [17]** |  | * | * |  | ** | * |  |  | 5* |
| **Urbino, 2023 [27]** | * | * | * | * |  | * | * | * | 7* |
| **Yeshurun, 2018 [18]** |  | * | * |  | ** | * | * | * | 7* |

**Supplementary Table 2**: Quality assessment of cohort studies included in the meta-analysis
